# Regulation of Translational Efficiency by Disparate 5′ UTRs of PPAR*γ* Splice Variants

**DOI:** 10.1155/2009/193413

**Published:** 2009-11-23

**Authors:** Shawn McClelland, Roopali Shrivastava, Jheem D. Medh

**Affiliations:** Department of Chemistry and Biochemistry, California State University Northridge, Northridge, CA 91330-8262, USA

## Abstract

The PPAR-*γ* gene encodes for at least 7 unique transcripts due to alternative splicing of five exons in the 5′-untranslated region (UTR). The translated region is encoded by exons 1–6, which are identical in all isoforms. This study investigated the role of the 5′-UTR in regulating the efficiency with which the message is translated to protein. A coupled *in vitro* transcription-translation assay demonstrated that PPAR-*γ*1, -*γ*2, and -*γ*5 are efficiently translated, whereas PPAR-*γ*4 and -*γ*7 are poorly translated. An *in vivo* reporter gene assay using each 5′-UTR upstream of the firefly luciferase gene showed that the 5′-UTRs for PPAR-*γ*1, -*γ*2, and -*γ*4 enhanced translation, whereas the 5′-UTRs for PPAR-*γ*5 and -*γ*7 inhibited translation. Models of RNA secondary structure, obtained by the mfold software, were used to explain the mechanism of regulation by each 5′-UTR. In general, it was found that the translational efficiency was inversely correlated with the stability of the mRNA secondary structure, the presence of base-pairing in the consensus Kozak sequence, the number of start codons in the 5′-UTR, and the length of the 5′-UTR. A better understanding of posttranscriptional regulation of translation will allow modulation of protein levels without altering transcription.

## 1. Introduction

Peroxisome proliferators-activated receptors (PPAR) are a family of nuclear receptors associated with cellular differentiation, and with the regulation of carbohydrate and lipid metabolism [1, 2]. PPAR consist of three main subtypes, PPAR-*α*, PPAR-*β* and PPAR-*γ*. Of these, PPAR-*γ* is the most extensively studied as it is implicated in several pathophysiological processes [3–5]. PPAR-*γ* are transcription factors that dimerize with the retinoid X receptor (RXR), and the heterodimers bind to specific DNA target sequences called PPAR response elements (PPREs) [6]. Numerous genes implicated in inflammation, cardiovascular disease, diabetes, and obesity are known to have a PPRE [7, 8]. Thus, the influence of PPAR on a cell is manifold and complex. 

**Figure 1:**
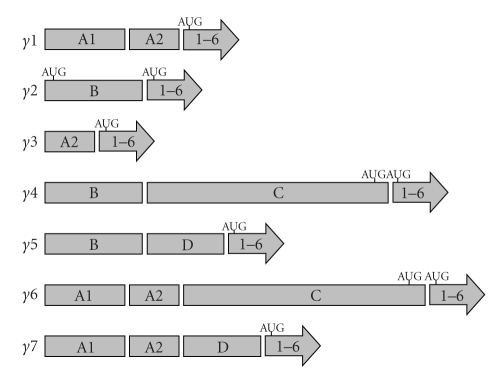
*Structures of *PPAR*γ splice variants.* Alignment of exons in seven PPAR*γ* mRNA splice variants is shown with 5′ end on the left and 3′ end on the right. The major translation initiation sites for each isoform are marked (ATG).

The PPAR-*γ* gene is found at chromosome 3p25 in humans [9]. Although transcription derives from only this one gene, several mRNA splice variants have been found [10, 11]. All splice variants consist of exons 1 through 6 consecutively on the 3′ end of the mRNA; these exons code for most of the actual PPAR-*γ* protein. The 5′ end of the mRNA consists of alternately spliced exons A1, A2, B, C, and D in various combinations to form seven splice variants. In each splice variant the exons at the 5′ end account for little or none of the final translated PPAR-*γ* protein. A schematic of the splice variants and their PPAR-*γ* protein start codons (ATG) can be seen in Figure 1.

The biological significance of the existence of multiple PPAR-*γ* transcript isoforms that encode for identical protein isoforms is not yet clear. The splice variants differ only in the 5′-UTR. It is likely that this region may contribute to posttranscriptional regulation of PPAR-*γ* protein expression. The 5′ UTR of apolipoprotein B was shown to increase the efficiency of translation in luciferase reporter gene assays and by *in vitro* translation assays [12]. The expression of serum amyloid A2 apolipoprotein was also posttranscriptionally regulated by both its 5′- and 3′-UTRs [13]. The translation of glutamate receptor 2 is inhibited by a polymorphic repeat sequence in its 5′-UTR [14]. Similarly, differences in their 5′-UTRs may influence the translational efficiency of PPAR-*γ* transcripts. 

There are several mechanisms by which the 5′-UTR may regulate translation. The presence of secondary stem-loop structures or short open-reading frames (ORFs) in the 5′-UTR considerably compromises translation efficiency [15]. Stable stem-and-loop structures in the 5′-UTR have been shown to block the migration of 40 S ribosomes during translation [16]. While moving along the transcript, the 40 S ribosomal subunit scans and evaluates initiation codons sequentially, starting at the 5′-end of the mRNA. The presence of short ORFs in the 5′-UTR allows the initiation complex to remain bound to the RNA even after wasteful translation of the short peptide. Thus, a small ORF greatly reduces but does not eliminate translation of the correct polypeptide [17, 18]. Other factors that affect posttranscriptional regulation of translation include the length of the 5′ UTR, and the sequence context of the initiation codon. 

In this study, we have investigated the influence of variable 5′-UTRs on the translation efficiency of PPAR-*γ* transcripts. All other variables being set equal, PPAR-*γ* makes for an excellent model to study the translational efficiency due to disparate 5′ UTR, but nearly identical translated regions. As for transcription factors, PPAR-*γ* may have a significant effect on non-mRNA-sequence elements involved in translation such as eIFs, ribosomes, and phosphorylation. However, the use of *in vitro* translation of specific splice variants excluded such factors from the equation, and any variability in translational efficiency could be attributed to the 5′ UTR. Additionally, the *in vivo* translational efficiency of each of the 5′-UTRs was compared using luciferase reporter gene assays [17, 19]. The experimental data were explained by *in silico* analysis of the whole transcript structures. 

As explained above, a primary mechanism for the regulation of translation is the formation of stem-loop secondary structures upstream of the initiation AUG [15]. The formation of mRNA secondary structure can be accurately predicted by computer programs that take into consideration mRNA sequence data, and the free energy change for the formation of various folded structures [18, 20]. The energy minimizing software mfold has been successfully used to investigate various areas of genomics. RNA folding software predicted how mutations in the 5′-UTR of hepatitis C virus RNA altered their stem-loop structure, thermodynamic stability, and binding affinity for ribosomal proteins [21]. The MFOLD software helped to identify a 35-nucleotide unfolded stretch in the 5′-UTR of the human cyclin dependent kinase inhibitor p27^Kip1^, indicating that this region may be the ribosomal recruitment site [22]. With the current efficacy of computer fold modeling, such analysis is a valuable tool in correlating translational efficiency of different PPAR-*γ* transcript isoforms to variations in mRNA secondary structure.

## 2. Materials and Methods

### 2.1. Preparation of Constructs with a T7 Polymerase Promoter.

A T7 promoter was an added upstream of full-length DNA splice variants for PPAR*γ*1, *γ*2, *γ*4, *γ*5, and *γ*7. This was accomplished by the PCR technique using previously cloned full-length genes, for each isoform, and specific primer sets. The sense primers were engineered to contain the T7 promoter sequence. For *γ*1 and *γ*7 the sense primer T7A1 (5′ TAA TAC GAC TCA CTA TAG GGC CTT TAC CTC TGC TGG TGA C 3′) was used. For the remaining splice variants the sense primer T7B (5′ TAA TAC GAC TCA CTA TAG GGA GCA AAC CCC TAT TCC ATG C 3′) was used. The same anti-sense primer (PPAR*γ*-antisense) was used for all splice variants (5′ CTA AAA CCG TTT CTT TTT AAA ATG C 3′) since they have identical 3′ ends. PCR was run using a 60°C annealing temperature and 40 cycles. A blank sample was created by using distilled water instead of a DNA template, the sense T7A1, and the antisense primers. 

The resulting products were resolved on a 1% agarose gel and displayed the expected sizes for the templates of interest (data not shown). The bands corresponding to each desired full-length splice variant with T7 promoter were excised (as well as the empty area where the band would be in the case of the blank) and DNA was extracted using a Geneclean II kit (QBiogene Irvine, CA). The amount of DNA was quantitated and the samples were stored in the −20°C freezer for future use in a linked *in vitro* transcription-translation reaction.

### 2.2. Linked In Vitro Transcription-Translation

Linked *in vitro* transcription-translation was performed using a Proteinscript II T7 kit from Ambion (Applied Biosystems, Foster City, CA). The kit allows coupled *in vitro* transcription and translation from a DNA template containing a T7 promoter upstream of the DNA to be transcribed. Equal amounts of gel-extracted DNA template (0.5 *μ*g of DNA for each template) for each splice variant were used for the reaction. The blank sample was used as a negative control and plasmid pTRI-Xef, provided with the kit, was used as a positive control. In one experiment, the pTRI-Xef template was mixed with DNA template for each splice variant to determine whether the DNA or mRNA of the specific splice variant inhibited translational efficiency of unrelated genes. The templates (6 *μ*L) were mixed with 2 *μ*L of 5X transcription mix and 2 *μ*L of T7 polymerase. The reaction was allowed to incubate at 30°C for 60 minutes and then placed on ice. At this time, the DNA templates had been transcribed into mRNA which could be run immediately in the *in vitro* translation step, or could be stored at −20°C for future use.

In vitro translation was carried out by first making a master mix of 24 *μ*L per each sample using 2 *μ*L nuclease free water, 1.25 *μ*L 20X translation mix, 1.25  *μ*L unlabeled methionine (500 *μ*M), 2 *μ*L [^35^S]-methionine, and 17.5 *μ*L retic lysate. To 24 *μ*L of the master mix, 1 *μ*L of the previously transcribed mRNA samples were added. The tubes were then gently mixed and incubated at 30°C for 60 minutes, after which they were immediately transferred to ice to stop the reaction. The products were now proteins with the incorporated radiolabeled methionine.

### 2.3. Analysis of In Vitro Translated Proteins

Analysis of the radiolabeled proteins was done by two different methods. First, a portion of the sample was precipitated using trichloroacetic acid (TCA). Briefly, 5 *μ*L of the translation product was mixed with 500 *μ*L of decolorizing solution (1 M NaOH, 1.5% H_2_O_2_, 1 mM L-Methionine) and 250 *μ*L of distilled water. The tubes were incubated for 10 minutes at 30°C followed by the addition of 1 mL of 25% TCA to precipitate the proteins. The pellets were dissolved in water and the amount of radioactivity was measured using a Beckman LS 6500 liquid scintillation counter. In another approach, the *in vitro* translation products were resolved by SDS-PAGE on a 12% acrylamide gel. The gel was fixed in a solution of 50% methanol and 7% acetic acid, washed, and soaked in a solution of 1 M sodium salicylate with 50% glycerol to enhance fluorographic detection of radiolabeled proteins. The gel was dried under vacuum at 80°C, and exposed to autoradiography film (Kodak BioMax MR) for one to four days at −80°C The films were developed using a Kodak RP X-OMAT Processor, scanned, and subjected to quantitative analysis of band intensity.

### 2.4. ImageJ Analysis

The relative abundance of *in vitro* translated proteins was determined by a densitometric analysis of the bands detected upon by autoradiography of the dried gels. An imaging software, ImageJ, available at the National Institutes of Health (NIH) website was used. Each band's intensity was quantitated by measuring the integrated density of a box made around each band and subtracting out the integrated density of the same sized box of the image's background. The band intensity of the darkest band was set to the value of one hundred and the intensities of other bands were assigned an adjusted relative value.

### 2.5. Dual Luciferase Reporter Gene Assay

The translational efficiency of each 5′-UTR was also measured through reporter gene constructs using the dual luciferase reporter (DLR) assay kit from Promega [17]. This method involves the cotransfection of chimeric firefly luciferase reporter gene constructs with a control construct expressing the *Renilla* luciferase gene. This allows for correction for any variation in transfection efficiency. The protocol described in the DLR assay kit was followed. For construction of the reporter gene vectors, the 5′ UTR of each of the different PPAR-*γ* transcript isoforms was PCR amplified using plasmids containing the corresponding full-length PPAR-*γ* DNA as templates, and specific primer pairs for each 5′-UTR, as shown in Table 1. The primers were designed with *Mlu I* and *Bgl II* restriction sites, to facilitate cloning into the pGL3-promoter luciferase reporter vector (Promega). After amplification, the PCR products were digested with Mlu I and Bgl II, gel purified, and cloned into the pGL3-promoter vector upstream of the firefly luciferase gene. Appropriate insertion of the 5′-UTR fragments was confirmed by sequencing from both ends using sequencing primers provided with the vector. The chimeric reporter gene (firefly luciferase) constructs were mixed with an expression vector for *Renilla* luciferase gene in a 9 : 1 mass ratio, and the two plasmids were transiently cotransfected into rat muscle cells (L6 cells) using Lipofectamine 2000. After 4 days, cell lysates were prepared and analyzed for levels of both firefly and *Renilla* luciferase activities using the DLR assay kit (Promega).

### 2.6. Statistical RNA Folding

The mfold server located at http://mfold.bioinfo.rpi.edu/ was used to model how the different splice variant mRNAs would fold [20]. Full sequences for each splice variant [11], obtained from the National Center for Biotechnology Information (NCBI), were analyzed using the Rensselaer Polytechnic Institute's web server running the mfold program. The mRNA sequences were simulated as though they were at 37°C in 1 M NaCl. Although physiological conditions typically maintain an ionic strength that is lower than 1 Molar,the conditions used in the simulation, 1 M NaCl is the current standard used in fold modeling. This standard is used in order to better compare modeling results between distinct experiments and simulations. Free energies and secondary structures for each splice variant were evaluated to discover possible correlations between experimental observations and stability and folding patterns of secondary structures. Other mRNA folding programs were also used to simulate mRNA secondary structures. In each case, nearly identical results were obtained, giving support to our mfold results.

## 3. Results and Discussion

### 3.1. In Vitro Transcription-Translation

Transfection studies with *in vivo* overexpression of PPAR*γ* splice variants suggested that they were translated with different efficiencies (data not shown). We, therefore, used an *in vitro* linked transcription-translation assay to examine the translational efficiencies of PPAR*γ* splice variants. Using this approach, we were able to examine the translational efficiency of each splice variant without regard to possible feedback inhibition or competition with other splice variants.

**Figure 2:**
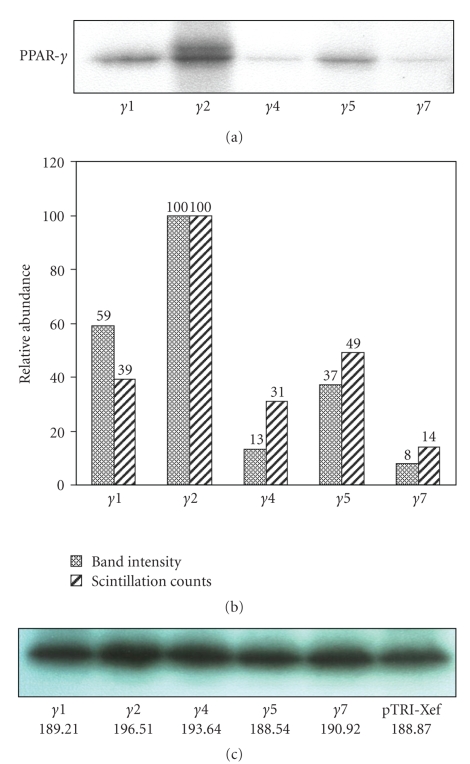
*In vitro Transcription-Translation.* Templates for the full-length PPAR-*γ*1, *γ*2, *γ*4, *γ*5, and *γ*7 splice variants were used to perform coupled *in vitro* transcription and translation reactions as described in “Methods”. Products were separated using SDS-PAGE. (a). The gel was dried and exposed to x-ray film to visualize *in vitro* labeled protein products. (b). *(dotted bars)* Band intensities were quantitated and the most optically dense band was set to 100. (b).* (hatched bars)* After the coupled *in vitro* transcription-translation reaction, labeled proteins were precipitated using TCA and the radioactivity in the precipitate was measured using a liquid scintillation counter. The highest counts were set to 100. (c). Templates for the *γ*1, *γ*2, *γ*4, *γ*5, and *γ*7 splice variants were mixed with a pTRI-Xef template and *in vitro* transcription and translation reactions were performed on each mixture. Bands were resolved by SDS-PAGE. The bands for pTRI-Xef are shown with the band intensities reported below each band.

Figure 2(a) shows results of a linked transcription-translation assay. Panel A is a representative autoradiogram of ^35^S-labeled proteins resolved by SDS-PAGE. It is evident that PPAR*γ*4 and PPAR*γ*7 are translated considerably less than PPAR*γ*2 and PPAR*γ*1. The translation of PPAR-*γ*5 was just slightly less than that of PPAR-*γ*1. The PPAR*γ*2 splice variant has two working start codons and translates into both the PPAR*γ*1 protein isoform and the PPAR*γ*2 protein isoform. The other splice variants translate to mainly the *γ*1 protein isoform. The PPAR*γ*4 transcript also has an additional initiation codon that would add 8 amino acids to the aminoterminus of the protein; however, such a protein isoform was not seen. The intensities for each band were quantitated using ImageJ (Figure 2(b)). The values are represented as a percent of the most intense band (resulting from the *γ*2 splice variant template) which was set to a value of 100. 

The products of the linked *in vitro* transcription-translation reaction were subjected to a TCA protein precipitation, and the radioactivity in the precipitated proteins was measured using a liquid scintillation counter (Figure 2(b)). Counts were adjusted such that the PPAR*γ*2 count equaled 100. The results corresponded closely to the SDS-PAGE-autoradiography values. Figure 2(b) shows data representative of two replicate independent experiments where the splice variants for PPAR-*γ*1 and PPAR-*γ*2 were consistently more efficiently translated than those for PPAR-*γ*4 and -*γ*7.

Our data indicated that the different 5′-UTRs of PPAR*γ* had a regulatory effect on the ability of the transcript to be translated. It was possible that the relatively lengthy 5′-UTRs of PPAR*γ*4 (389 bases) and -*γ*7 (240 bases) could sequester the translation machinery of the cell and impose a broad repression of translation of all cellular transcripts. To examine this possibility, the DNA templates of each PPAR*γ* splice variant were mixed with a reference DNA template, pTRI-Xef, that was supplied with the Proteinscript II kit (Ambion). Results of linked *in vitro* transcription-translation using the mixed templates, as well as of standard pTRI-Xef alone, are shown in Figure 2(c). After SDS-PAGE, the intensities of all samples were quantified and are shown below the corresponding bands, but they fell within 5% of each other. This experiment shows that templates or transcripts for any of the PPAR-*γ* splice variants do not slow down translation of an unrelated protein in an *in vitro* assay.

### 3.2. In Vivo Reporter Gene Assays

**Figure 3:**
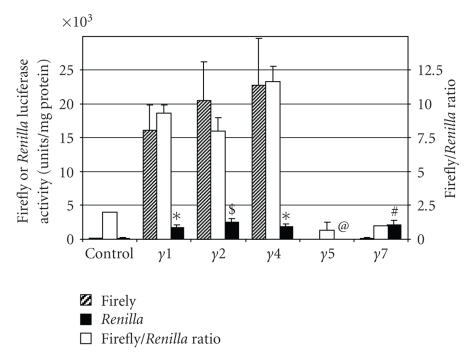
*In vivo reporter gene assays.* Firefly luciferase reporter gene constructs were designed to contain individual 5′-UTRs upstream of the luciferase reporter gene in the pGL3 vector (Promega). Rat L6 cells were transfected with either the host pGL3 vector (control) or the vector containing a specific PPAR-*γ* 5′-UTR. Cells were cotransfected with a plasmid expressing the *Renilla* luciferase enzyme. After 4 days, cell lysates were prepared and analyzed for firefly (hatched bars) and *Renilla* (solid bars) luciferase activities. The open bars show the ratio of firefly to *Renilla* luciferase (*n* = 3, *P* values relative to control are ∗ <.005, # <.05, *$* <.01, @ <0.5).

Next, we studied regulation of *in vivo* translation by each of the PPAR-*γ* 5′-UTRs using a luciferase reporter gene assay. When equal amounts of 5′-UTR-firefly luciferase gene constructs were transfected into cultured L6 cells (an easily transfected rat muscle cell line), and cell lysates corrected for equal protein mass were used, the expression of luciferase activity was significantly enhanced compared to control (pGL3 vector without any insert upstream of the luciferase gene) when the 5′-UTRs for PPAR-*γ*1, -*γ*2, and *γ*4 were inserted upstream of the firefly luciferase reporter gene (Figure 3, hatched bars). The firefly luciferase activity was not altered relative to control when the 5′-UTR for PAPR-*γ*7 was inserted upstream of the reporter gene, whereas it was repressed and was undetectable when the 5′-UTR of PPAR-*γ*5 was inserted in the vector. The activity for the cotransfected *Renilla* luciferase was much lower than the firefly luciferase (Figure 3, solid bars). This was consistent with the transfection ratio of 9 : 1 for firefly: *Renilla* luciferase plasmids. Control experiments with purified firefly luciferase and *Renilla* luciferase enzymes confirmed that mixing the two enzymes did not interfere with the quantitative measurement of individual enzyme activities (data not shown). The *Renilla* luciferase activity was several fold greater than control in cells cotransfected with firefly luciferase constructs containing the 5′-UTRs for PPAR-*γ*1, PPAR-*γ*2, PPAR-*γ*4, and PPAR-*γ*7, but it was undetectable in cells cotransfected with PPAR-*γ*5 5′-UTR-luciferase contructs. Since the *Renilla* luciferase served as a transfection control, results are also expressed as a ratio of the firefly luciferase to the *Renilla* luciferase activities (Figure 3, open bars, *n* = 3, *P* values are shown), as is customary for the DLR assay [17]. The ratio of the two enzymes was the highest for cells transfected with the 5′-UTRs for PPAR-*γ*4, PPAR-*γ*1, and PPAR-*γ*2. The results for the *in vivo* DLR assay suggest that the presence of these three 5′-UTRs enhance translation, whereas the 5′-UTRs for PPAR-*γ*5 and PPAR-*γ*7 repress translation of the firefly luciferase gene, compared to control. Interestingly, even though the expression of *Renilla* luciferase activity is used as a control for transfection efficiency, and is expected to vary randomly, its expression showed the exact pattern in multiple experiments, with the level being relatively high (compared to control) when cotransfected with reporter gene constructs containing 5′-UTRs of PPAR-*γ*1, -*γ*2, -*γ*4, or -*γ*7. The presence of the PPAR-*γ*5 5′-UTR always failed to stimulate *Renilla* luciferase activity. Since the *Renilla* luciferase gene is itself not driven by any variable cis-acting upstream elements in the different transfections, our results suggest that perhaps the 5′-UTRs inserted in the cotransfected reporter gene constructs may be influencing the translation of *Renilla* luciferase in a transacting manner. This is contrary to the absence of any trans regulation in the *in vitro* assay (Figure 2(c)); however, the presence of cellular elements in the *in vivo* DLR assay may contribute factors that may bind to and regulate the activities of both the *Renilla* and firefly luciferase enzymes. It may be interesting to investigate whether this trans-regulation is different in different cell types.

### 3.3. RNA Fold Modeling

In order to explain differences in translational efficiency for each splice variant, we examined differences in the primary and secondary structures of each transcript variant. Recent advances in computational modeling of DNA and RNA have made such an investigation a viable approach. 

Using the mfold RNA-folding software, each splice variant's full mRNA sequence was computationally folded. The mfold software reports several folded structures along with the free energy change of folding. In order to predict the average stability of secondary structures, mfold also calculates the probability of interactions between any two bases in the input sequence. It prepares an energy dot plot, where each dot represents a possible base pair formation and a chain of dots represents possible helical structures. Such energy plots were much denser for PPAR-*γ*4, PPAR-*γ*5, and PPAR-*γ*7 compared to those for PPAR-*γ*1 and PPAR-*γ*2 (not shown) indicating the lower stability of secondary structures for PPAR-*γ*1 and PPAR-*γ*2. Table 2 shows the optimum free energy change for secondary structure formation computed by such energy dot plots. 

**Figure 4:**
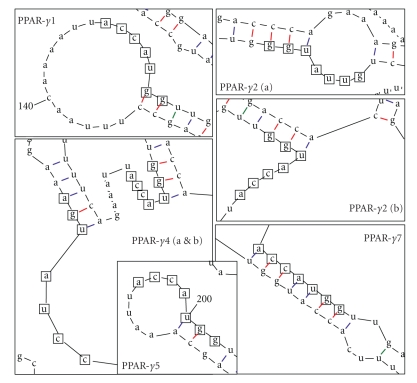
*Magnified RNA folding Models.* Full-length PPAR-*γ* splice variants were folded using the mfold software. The start codon regions for each isoform are magnified here to show the canonical start codon region (accAUGg) and the number of bases in that region that are bound.

The most stable structures with the lowest free energy change were used to magnify the start codon regions for each of the splice variants (Figure 4). Closer inspection of secondary structures reveals possible indicators of each splice variant's translational efficiency. Some factors that are known to reduce translation efficiency are longer 5′-UTRs with multiple start codons that may result in false starts or short ORF segments that lead to nonsense products [15]. Both PPAR*γ*1 and PPAR*γ*2 splice variants translated efficiently due to the fact that their secondary structures are the least stable, they have the shortest 5′-UTRs, and the fewest start codons in their 5’-UTRs (Table 2). The PPAR*γ*7 splice variant also has only 1 start codon in its 5′-UTR, but it is very poorly translated. To explain this, we examined the sequence around the start codon more closely. The ribosome of the translation initiation complex recognizes a Kozak consensus sequence at the translation start site [23]. In Figure 4, this region accAUGg is highlighted and the number of consensus bases that are bound in the secondary structure is reported in Table 2. For PPAR*γ*7, all seven bases of the start motif completely match the Kozak sequence, and each base in the region is bound in a secondary structure. This may be the reason why this splice variant is translated very inefficiently during the *in vitro* and *in vivo* translation experiments. Similarly, the upstream alternative start codons in PPAR-*γ*2 and PPAR-*γ*4 (indicated as PPAR-*γ*2 b and PPAR-*γ*4 b in Figure 4) have a weaker consensus motif and this may be the reason why the larger size proteins were not efficiently translated in Figure 2. The *in vitro* experiment (Figure 2) is also consistent with the predicted translational efficiency for PPAR-*γ*4 and PPAR-*γ*5. PPAR-*γ*4 is most inefficiently translated due to the finding that its secondary structure is most stable (lowest ∆G° values), and it has the longest 5′-UTR with 12 putative start codons. On the other hand, translation of PPAR-*γ*5 is intermediate, since none of the inhibitory factors are extreme. The *in vivo* experiments with luciferase reporter gene assays indicate that translational efficiency of PPAR-*γ*4 is very efficient. This could be due to the presence of cellular factors that may bind to the 5′-UTR and promote translation. Certain nucleotide sequences in the 5′-UTR may form secondary structures that can function as internal ribosome entry sites (IRES) [24]. These structures require additional cellular proteins called IRES trans-acting factors (ITAFs) to promote translation [25]. Since ITAFs would be present in the *in vivo* model but absent in the *in vitro* translation, it explains why the PPAR-*γ*4 5′-UTR drives translation *in vivo* but not *in vitro*.

## 4. Conclusion

The reason for the existence of splice variants is a source of much speculation. The foremost theory is that it provides greater flexibility and diversity of protein expression without the need for more DNA. However, in the case of PPAR*γ*, the splice variants seem to play a different role. As opposed to producing varying proteins, they produce essentially the same protein. Instead, the PPAR*γ* splice variants appear to regulate protein expression. While other mechanisms of regulation may also contribute, a primary mechanism appears to be the differing translational efficiencies of the many splice variants. 

In an attempt to explicate what is causing the differences in translational efficiency, we turned to RNA secondary structure modeling. Previous studies have attributed 5′-UTR length and number of start codons as key factors in changing translational efficiencies of mRNA strands [26, 27]. However, these factors alone did not seem to completely explain translation of the PPAR*γ* splice variants. Length and number of start codons are both attributes related to the primary sequence of the RNA. Looking at secondary structure gave a better insight and understanding of the role of 5′-UTRs in regulation of translation. As research progresses in the area of mRNA secondary structure and its interactions, we will be even better at predicting translational efficiencies. For instance, as the ability to identify riboswitch sequences increases, we would be able to determine whether different riboswitches present in different splice variants influence translational efficiency.

As we better understand the regulatory functions of each PPAR-*γ* splice variant, it will become possible to modulate PPAR-*γ* protein expression and therefore its end cellular effect. This will lead to better treatments and management of countless diseases that PPAR-*γ* is implicated in.

## 5. Acknowledgments

This work was supported by a National Institutes of Health MBRS-SCORE Award (S06 GM8680) and a National Institutes of Health AREA Grant (R15 HL083946). We thank Kumuda Saraff for helpful discussions and assistance at the bench.

## Figures and Tables

**Table 1:** Primers for amplification of different PPAR-*γ* 5′-UTRs.

5′ UTR of	Sense primer	Antisense primer	Size (bp)
PPAR-*γ*1	ATCACGCGTCCTTTACCTCTGCTGGTGACA	CGGAGATCTTGTTAAAGGCTGACTCTTGTT	177
PPAR-*γ*2	GTAACGCGTAGCAAACCCCTATTCCATGCT	CGGAGATCTCTTGTGATATGTTTGCAGACA	142
PPAR-*γ*4	GTAACGCGTAGCAAACCCCTATTCCATGCT	CGGAGATCTCTGAAAAGCCTTTCATAGGTC	405
PPAR-*γ*5	GTAACGCGTAGCAAACCCCTATTCCATGCT	CGAAGATCTTAATCCCAGCACTTTGGGAGG	221
PPAR-*γ*7	ATCACGCGTCCTTTACCTCTGCTGGTGACA	CGAAGATCTTAATCCCAGCACTTTGGGAGG	256

**Table 2:** Comparison of structural elements of 5′-UTRs of PPAR-*γ* transcript isoforms.

Splice variant	No. of start codons	No. of start codons in 5′-UTR	Length of 5′-UTR (bases)	Strength of Kozak sequence (No. of matching bases)	No. of Kozak sequence bases bound in secondary structure	∆G° from energy dot plots kCal/mol
*γ*1	37	0	141	7	2	−516
*γ*2	39	2	106	4, 7	3, 3	−507
*γ*4	49	12	369	4, 7	3, 3	−590
*γ*5	40	3	190	7	2	−543
*γ*7	38	1	225	7	7	−547
